# Decreased antibody response to influenza vaccine with an enhanced antibody response to subsequent SARS‐CoV‐2 vaccination in patients with chronic hepatitis B virus infection

**DOI:** 10.1002/iid3.759

**Published:** 2022-12-31

**Authors:** Taiyu He, Ning Ling, Gaoli Zhang, Dejuan Xiang, Peng Hu, Mingli Peng, Dachuan Cai, Dazhi Zhang, Min Chen, Hong Ren

**Affiliations:** ^1^ Department of Infectious Diseases, Key Laboratory of Molecular Biology for Infectious Diseases (Ministry of Education), Institute for Viral Hepatitis, The Second Affiliated Hospital Chongqing Medical University Chongqing China

**Keywords:** CHB, cirrhosis, influenza, SARS‐CoV‐2, immune response

## Abstract

**Introduction:**

Influenza or SARS‐CoV‐2 vaccination is especially recommended for people with underlying diseases. For the large number of patients with chronic hepatitis B virus infection (CHB), studies on their immune responses to these vaccines are still lacking.

**Methods:**

A total of 57 CHB patients and 19 healthy controls (HCs) receiving inactivated influenza vaccination were prospectively followed up. Influenza‐specific immunoglobulin G (IgG) antibodies (anti‐H1N1, anti‐H3N2, and anti‐B IgG), antibody‐secreting cells (ASCs), and circulating T follicular helper cells were assessed simultaneously. Eight CHB patients subsequently got inactivated SARS‐CoV‐2 vaccination during 1‐year follow‐up, and levels of serum antibodies against SARS‐CoV‐2 were further analyzed.

**Results:**

On day 28 after influenza vaccination, three influenza antibodies levels appeared to be lower in CHB patients than in HCs. And anti‐H1N1 IgG level was significantly decreased in cirrhotic patients (*p* < .05). Anti‐H1N1 IgG levels (day 28) were positively correlated with ASC frequencies (day 7) (*p* < .05), and negatively correlated with cirrhosis and hepatitis B surface antigen levels (*p* < .05). Anti‐SARS‐CoV‐2 antibodies were higher in patients with influenza vaccination history than in patients without the history (*p* < .05). Moreover, positive correlations existed between influenza vaccination history and anti‐SARS‐CoV‐2 antibody levels (*p* < .01).

**Conclusions:**

CHB patients, especially those with cirrhosis, appeared to have a decreased antibody response to inactivated influenza vaccine. A history of inactivated influenza vaccination within 1 year before inactivated SARS‐CoV‐2 vaccination might induce stronger anti‐SARS‐CoV‐2 antibody response.

## Graphical abstract

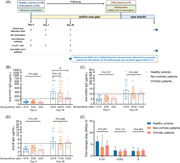

## 1 INTRODUCTION

Annual influenza epidemics cause approximately 3–5 million cases of severe illness and about 290,000–650,000 respiratory deaths globally.^1^, ^2^ Influenza vaccination is important every year but even more so in these few years as we are facing multiple risks of influenza and the coronavirus disease 2019 (COVID‐19) at the same time.

A decline in immune responses to flu or COVID‐19 vaccination was found in persons with underlying medical conditions, such as obesity,^3^ cancer,^4^ or immunocompromised diseases.^5^ Apart from these well‐defined chronic diseases which are strongly related to weak immune response to vaccination, there is evidence that some other medical conditions (such as chronic infection) may also contribute to reduced vaccine responses.^6^, ^7^, ^8^ For example, patients with chronic hepatitis C virus are reported to have weaker immune responses to vaccines,^7^, ^8^ which might weaken the immune protection provided by vaccination.

Chronic hepatitis B virus infection (CHB) remains a major global public health concern and is closely associated with severe clinical complications, especially in eastern Asia.^9^ Persistent hepatitis B virus infection can lead to not only chronic liver damage, but also immune imbalance or chronic inflammation.^10^, ^11^ The impact of CHB on immune responses to vaccine is unclear yet. Our latest data indicated that CHB patients had lower anti‐SARS‐CoV‐2 antibody titers than healthy controls (HCs) at 1 month after inactivated SARS‐CoV‐2 vaccination.^12^

Therefore, this study continues to evaluate humoral immune responses to flu vaccination in CHB patients. In addition, recent studies have reported that influenza vaccination affects the cytokine response to SARS‐CoV‐2 ^13^ and may reduce infection, severe symptoms, and death caused by this virus.^14^, ^15^, ^16^ Hence, we further assess the anti‐SARS‐CoV‐2 antibody titers in these follow‐up CHB patients who received inactivated SARS‐CoV‐2 vaccine within 1 year after influenza vaccination.

## 2 MATERIALS AND METHODS

### 2.1 Participant enrollment

From October 12, 2020, to January 13, 2021, 57 adult patients with CHB and 19 healthy adults as controls were enrolled at the Second Affiliated Hospital of Chongqing Medical University. The inclusion criterion for CHB patients was hepatitis B surface antigen (HBsAg) positivity for more than 6 months. The inclusion criteria for the HCs were HBsAg‐negativity and no self‐reported or documented disease. For all participants, the following conditions were excluded: (1) history of laboratory‐confirmed influenza virus infection; (2) history of hospitalization for respiratory symptoms during influenza seasons; (3) hepatitis C virus/human immunodeficiency virus infection; (4) malignant tumor, renal failure, or other major diseases; (5) pregnancy; and (6) use of immunosuppressants.

All participants were intramuscularly vaccinated with one dose of an inactivated, split‐virion, quadrivalent influenza vaccine (components: A/Guangdong‐Maonan/SWL1536/2019 (H1N1) pdm09‐like virus, A/Hong Kong/2671/2019 (H3N2)‐like virus, B/Washington/02/2019 (B/Victoria lineage)‐like virus, B/Phuket/3073/2013 (B/Yamagata lineage)‐like virus, HUALAN BIO). Furthermore, 8 CHB patients subsequently received two doses (full course) of an inactivated SARS‐CoV‐2 vaccine within 1 year after the influenza vaccination.

To obtain the controls who had received only inactivated SARS‐CoV‐2 vaccine without influenza vaccination, we retrospectively reviewed the records of anti‐SARS‐CoV‐2 antibody data in the physical examination center from August to October 2021. Finally, data were collected from propensity‐matched 64 CHB patients and 46 healthy people as the controls. These controls were further confirmed to have full course of inactivated SARS‐CoV‐2 vaccine while no history of influenza virus infection and influenza vaccination within the last year.

This study was approved by the ethics committee of the Second Affiliated Hospital of Chongqing Medical University and registered at ClinicalTrials.gov (NCT05007665, date: August 16, 2021) and conformed to the ethical guidelines of the Declaration of Helsinki. Written informed consent was obtained from all participants before enrollment.

### 2.2 Data and sample collection

Demographic and clinical data were obtained using questionnaires and electronic medical records. Adverse effects (AEs) were determined using questionnaires, verified by investigators, and graded according to the scale issued by the National Medical Products Administration of China (version 2019).

Blood samples were collected from all participants before (day 0) and on days 7 and 28 after influenza vaccination. Levels of antiinfluenza antibodies were determined on days 0 and 28. Anti‐SARS‐CoV‐2 antibody levels were evaluated on day 28. Percentages of B and T cells, and laboratory parameters (routine blood test, liver function test, serum HBV markers and HBV DNA) were detected on days 0, 7, and 28. The laboratory parameters were detected by the department of the clinical laboratory of the Second Affiliated Hospital, Chongqing Medical University.

For the 8 CHB patients who got subsequent SARS‐CoV‐2 vaccination, blood samples were drawn at 1 month after full SARS‐CoV‐2 vaccination for detection of anti‐SARS‐CoV‐2 antibodies. Anti‐SARS‐CoV‐2 antibodies data were retrospectively collected in those 64 CHB patients and 46 healthy people who were only vaccinated against SARS‐CoV‐2 at 1 month after full vaccination.

**Figure 1:**
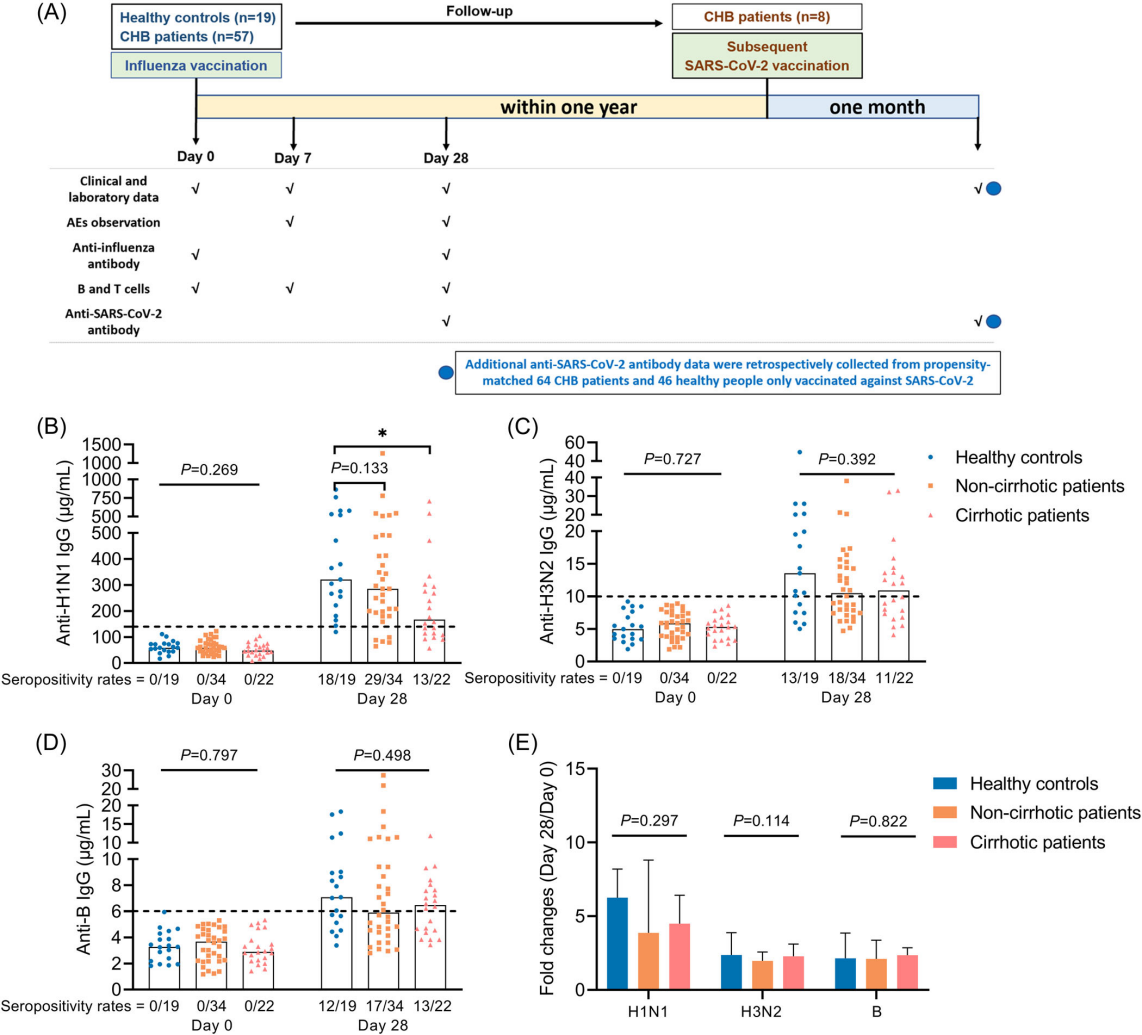
Study flow chart and antibody response to influenza vaccine. (A) Study flow chart. (B–D) Anti‐H1N1 IgG (B), anti‐H3N2 IgG (C), and anti‐B IgG (D) levels in HCs, cirrhotic and non‐cirrhotic patients with CHB at day 0 and 28. (E) Fold changes of antibody levels (day 28/day 0) in HCs, cirrhotic and non‐cirrhotic patients with CHB. Kruskal–Wallis test was used for comparison. Bonferroni's correction was used, and adjusted *p* values were represented in this figure. Top of all bars represents median value. *p < .05. Day 0 represents the influenza vaccination day, day 7 represents 7 days after influenza vaccination, and day 28 represents 28 days after influenza vaccination. HCs, healthy controls; IgG, immunoglobulin G.

The study flow chart is shown in Figure 1A.

### 2.3 Anti‐influenza antibody evaluation by enzyme‐linked immunosorbent assay (ELISA)

Levels of anti‐influenza‐H1N1 immunoglobulin G (IgG) (anti‐H1N1 IgG), anti‐influenza‐H3N2 IgG (anti‐H3N2 IgG) and anti‐influenza‐B IgG (anti‐B IgG) were determined using ELISA kits (15500/15503/SD‐10000D, Lun Changshuo Biological Technology, China) according to the manufacturer's instructions. Briefly, H1N1 (A/Guangdong‐Maonan/SWL1536/2019 (H1N1) pdm09‐like virus)/H3N2 (A/Hong Kong/2671/2019 (H3N2)‐like virus)/B (B/Phuket/3073/2013(B/Yamagata lineage)‐like virus) solid‐phase antigen was precoated onto 96‐well plates. Serially diluted standards (50 μl/well) and samples (50 μl/well) were added and incubated at 37°C for 30 min. ELISA was performed in duplicate. After washing the plates five times with washing solution (phosphate‐buffered saline [PBS] + 0.075‰ Tween 20), horseradish peroxidase (HRP)‐conjugated goat anti‐human IgG was added (100 μl/well) and incubated at 37°C for 30 min. After five washes, the substrate solution (3,3′,5,5′‐tetramethyl benzidine, TMB) was added (100 μl/well) and incubated at 37°C for 15 min. The stop solution was then added (50 μl/well), and optical density (OD values) at 450 nm was measured using a microplate reader (RT6100; Rayto). Finally, IgG concentrations (μg/ml) were calculated using a standard curve. The cutoff values for anti‐H1N1 IgG, anti‐H3N2 IgG, and anti‐B IgG were 140 μg/ml, 10 μg/ml, and 6 μg/ml, respectively.

### 2.4 Assay for anti‐RBD IgG and NAb against SARS‐CoV‐2

Serum anti‐receptor‐binding‐domain IgG (anti‐RBD IgG) and neutralizing antibody (NAb) were evaluated using capture chemiluminescence immunoassays by MAGLUMI X8 (Snibe) following the manufacturer's instructions. The sensitivity and specificity of the kits (Snibe) for anti‐RBD IgG were 100% and 99.6%, respectively, and those for NAb were 100% and 100%, respectively. The cutoff values were 1.00 AU/ml for anti‐RBD IgG and 0.15 μg/ml for NAb.

### 2.5 Detection of antibody‐secreting cells (ASCs) and cTFH cells by flow cytometry

Heparinized whole blood was collected at three time points (days 0, 7, and 28) to detect ASCs and circulating T follicular helper (cTFH) cells. Two milliliters of 1× RBC Lysis Buffer (420301; BioLegend) was added to each tube containing 100 µl of whole blood. After incubation at room temperature (RT) in the dark for 10 min, the solution was centrifuged at 400*g* for 5 min. The supernatant was aspirated without disturbing the pellet, and 5 μl Human TruStain FcX™ (422302; BioLegend) was added for Fc receptor blocking. After incubation at RT protected from light for 10 min, conjugated antibodies were added for 30 min of staining. For ASC staining, PerCP/Cyanine 5.5 anti‐human CD3 (1:50, 300430; BioLegend), APC anti‐human CD19 (1:50, 302212; BioLegend), Brilliant Violet 605™ anti‐human CD20 (1:50, 302334; BioLegend), PE anti‐human CD27 (1:50, 356406; BioLegend), and PE/Cyanine 7 anti‐human CD38 (1:50, 303516; BioLegend) antibodies were used. For cTFH cell staining, PerCP/Cyanine5.5 anti‐human CD3 (1:50, 300430; BioLegend), FITC anti‐human CD4 (1:50, 300506; BioLegend), Brilliant Violet 421™ anti‐human ICOS (1:50, 313524; BioLegend), APC anti‐human CXCR3 (1:50, 353708; BioLegend), PE anti‐human CXCR5 (1:50, 356904; BioLegend), and Brilliant Violet 605™ anti‐human CCR6 (1:50, 353420; BioLegend) were added. After staining, the cells were washed and resuspended in 200 µl FACS buffer (PBS supplemented with 2% fetal bovine serum [FSD500; Excell Bio]).

Approximately 1 × 10^5^ events were collected within a lymphocyte gate by flow cytometry (CytoFLEX; Beckman Coulter). FlowJo (10.0.7r2; Treestar) was used for analysis of the cell populations. The full gating strategies are provided in Figure S1.

### 2.6 Enzyme‐linked immunosorbent assay (ELISPOT) for H1N1‐specific IgG^+^ peripheral blood mononuclear cell (PBMCs)

H1N1‐specific IgG^+^ PBMCs were detected using the ELISPOT kit (CT780‐PR5; U‐CyTech Biosciences) according to the manufacturer's protocol. Briefly, 96‐well plates (MSHAN4B50; Millipore) were coated overnight at 4°C with either 4 μg/ml influenza A H1N1 (A/Guangdong‐Maonan/SWL1536/2019) hemagglutinin protein (40717‐V08H; Sino Biological) or with a coating antibody (50 μl/well). The assay was performed in duplicate. After washing with PBS, the plates were blocked by incubation with blocking buffer (200 μl/well) at RT for 1 h. After removing the blocking solution, approximately 0.5 × 10^5^ PBMCs isolated from heparinized whole blood using Histopaque (10771; Sigma–Aldrich) according to the manufacturer's instructions were added (100 μl/well) and incubated overnight at 37°C and 5% CO_2_. After washing with PBS and wash buffer, diluted biotinylated detection antibody was added (100 μl/well) and incubated at RT for 2 h. The plates were again washed (wash buffer), and diluted streptavidin‐HRP conjugate was added (100 μl/well) and incubated at RT for 2 h. After washing with wash buffer, the AEC substrate solution was added (100 μl/well) and incubated at RT for 30 min. After washing and air‐drying, the plates were analyzed using an ELISPOT reader (AID, ELR08 IFL). The ELISPOT assay image for H1N1‐specific IgG^+^ PBMCs is shown in Figure S2.

### 2.7 Statistical analysis

The Chi‐square and Fisher's exact tests were used for comparison of categorical variables. For continuous‐variable comparisons, the Wilcoxon signed‐rank test (two groups) and Friedman test (three or more groups) were used for paired groups, and the Mann–Whitney *U* test (two groups) and Kruskal–Wallis test (three or more groups) were used for unpaired groups. All results of multiple comparisons were corrected using Bonferroni's correction, and the *p* values shown in figures were all adjusted. For correlation analysis, Spearman's rank correlation was used when two variables were continuous; otherwise, Kendall's rank correlation was employed. Multivariate linear regression analysis was conducted to obtain factors that significantly affected antibody titers. A two‐sided *p* < .05 was considered statistically significant. SPSS (24.0.0; IBM) was utilized for statistical analysis and GraphPad Prism (9.2.0; GraphPad Software Inc.) for plotting.

## 3 RESULTS

### 3.1 Participant characteristics and influenza vaccination safety

In this study, 57 CHB patients and 19 HCs had similar ages, sex ratios, and body mass indices. In 57 CHB patients, apart from 8 patients with mildly elevated levels of ALT and AST (between one to two times the upper limit of normal [2×ULN]), the rest had normal liver functions and routine blood tests. Twenty‐two CHB patients were diagnosed with compensated liver cirrhosis based on guidelines,^17^ and a significantly lower platelet count was found in these cirrhosis patients. Detailed baseline clinical and laboratory data of the 19 HCs, 35 CHB patients without cirrhosis, and 22 CHB patients with cirrhosis are listed in Table 1 and Table S1.

**Table 1:** Characteristics of healthy controls and patients with CHB vaccinated against influenza

Variables	Healthy controls (*n* = 19)	Patients (*n* = 57)	*p* value
Age^a^ (years)	51 (24–62)	52 (25–67)	.652
18–60, *n* (%)	17 (89.5%)	45 (78.9%)	.494
≥60, *n* (%)	2 (10.5%)	12 (21.1%)	
Gender			
Male, *n* (%)	9 (47.4%)	32 (56.1%)	.506
Female, *n* (%)	10 (52.6%)	25 (43.9%)	
BMI^a^ (kg/m^2^)	23.69 (19.05–28.08)	24.22 (18.52–31.57)	.381
RBC^a^ (10^9^/L)	4.48 (3.98–5.77)	4.57 (3.68–5.76)	.408
PLT^a^ (10^9^/L)	187 (78–321)	157 (39–302)	**.024**
WBC^a^ (10^9^/L)	6.86 (3.64–10.55)	5.84 (1.38–12.14)	.102
Lymphocyte^a^ (10^9^/L)	1.98 (1.15–3.26)	1.75 (0.28–3.00)	.083
ALT^a^ (U/L)	19 (9–49)	25 (8–95)	**.038**
AST^a^ (U/L)	20 (13–38)	25 (15–76)	**.001**
HBsAg			
<250 IU/ml, *n* (%)	–	32 (56.1%)	–
≥250 IU/ml, *n* (%)	–	25 (43.9%)	
HBeAg [positive, *n* (%)]	–	12 (21.1%)	–
HBV DNA^a^ (IU/ml)	–	100 (20–1.75 × 10^8^)	–
Antiviral treatment, *n* (%)	–	41 (71.9%)	–
Liver cirrhosis, *n* (%)	–	22 (38.6%)	–

Abbreviations: ALT, alanine aminotransferase; AST, aspartate aminotransferase; BMI, body mass index; CHB, chronic hepatitis B virus infection; HBeAg, hepatitis B e antigen; PLT, platelet; RBC, red blood cell; WBC, white blood cell.

^a^ Presented as median (range). The Chi‐Square statistic was used for categorical variables, and Mann–Whitney *U* test was used for continuous variables.

AEs that occurred in patients within 7 and 28 days after influenza vaccination are shown in Table S2. The incidences of AEs were similar in the patients without cirrhosis, the patients with cirrhosis and HCs, and all AEs were mild or moderate (grade 1 or 2).

Levels of routine blood test parameters, liver function indices and HBV DNA were compared before and after influenza vaccination (Table S3). Although levels of several laboratory parameters exhibited statistically significant changes in CHB patients after influenza vaccination (*p* < .01), these changes were all mild. Those CHB patients with baseline abnormal ALT and AST levels did not show significantly increased ALT or AST levels after vaccination.

### 3.2 Antibody responses to influenza vaccine

On day 0, anti‐H1N1 IgG, anti‐H3N2 IgG and anti‐B IgG were negative in all patients and HCs (Figure 1B–D). On day 28 postvaccination, levels of all three antibodies had increased significantly in both patients and HCs (Figure S3). And the seropositivity rates were similar between all CHB patients and HCs (anti‐H1N1 IgG: 75.0% vs. 94.7%, *p* = .127; anti‐H3N2 IgG: 51.8% vs. 68.4%, *p* = .207; anti‐B IgG: 53.6% vs. 63.2%, *p* = .467). But the antibody levels appeared to be lower in patients with CHB (anti‐H1N1 IgG: 244.1 vs. 321.4, *p* = .044; anti‐H3N2 IgG: 10.5 vs. 13.5, *p* = .172; anti‐B IgG: 6.3 vs. 7.1, *p* = .252) (Figure S3). Notably, anti‐H1N1 IgG levels were significantly lower in patients with cirrhosis than in HCs (167.2 vs. 321.4, *p* = .017) at day 28 (Figure 1B). In addition, after influenza vaccination, fold changes of anti‐H3N2 IgG levels were lower in all CHB patients than in HCs (*p* = .042) (Figure S3D), whereas those of anti‐H1N1 IgG and anti‐B IgG levels were similar in patients and HCs (Figure 1E and Figure S3D).

### 3.3 ASC and cTFH responses to influenza vaccination

**Figure 2:**
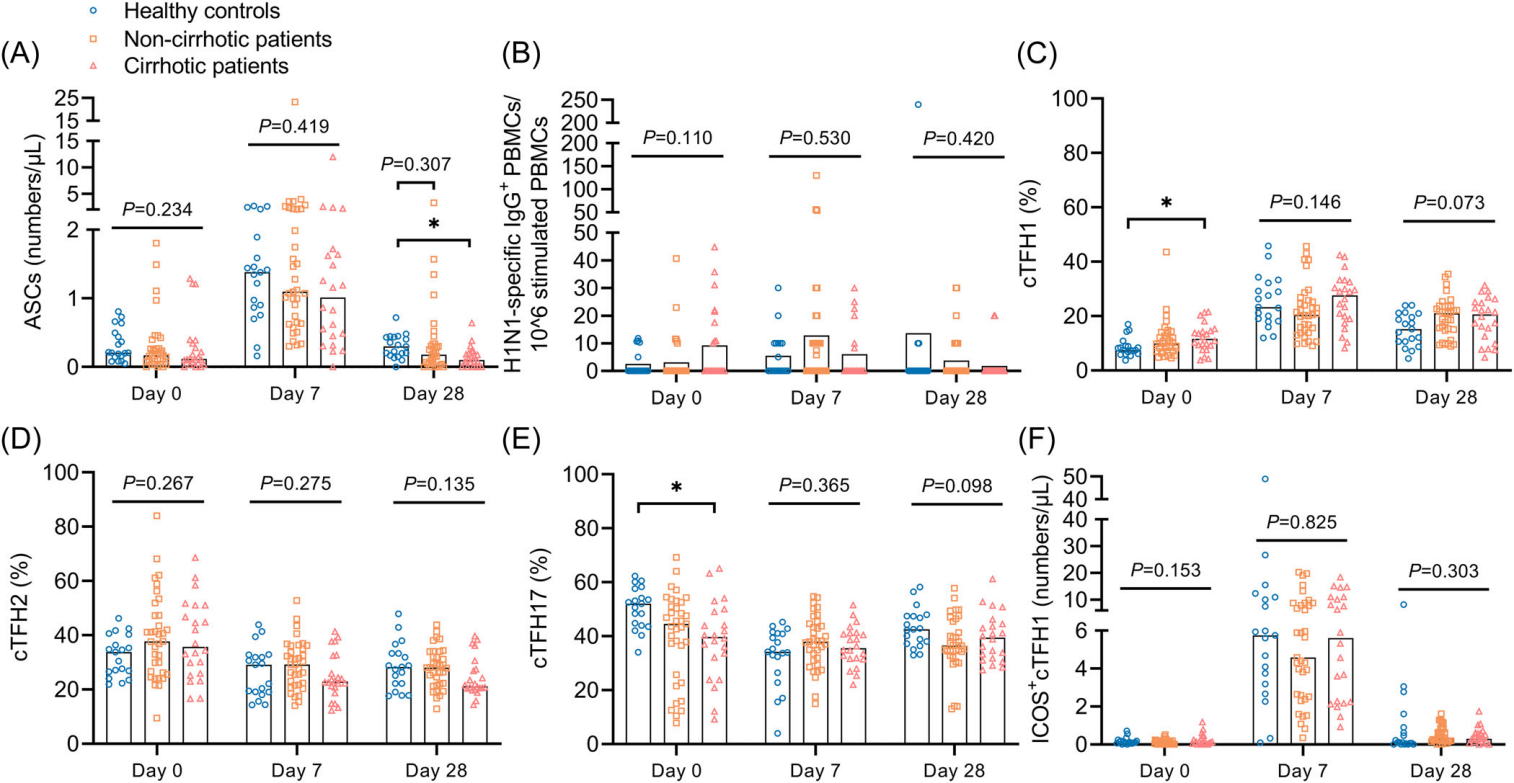
Cell response to influenza vaccine in healthy controls, cirrhotic and non‐cirrhotic patients with CHB. (A–F) Frequencies of ASCs (A), H1N1‐specific IgG^+^ PBMCs (B), cTFH1 (C), cTFH2 (D), cTFH17 (E), and ICOS^+^cTFH1 (F) cells in healthy controls, cirrhotic and non‐cirrhotic patients with CHB at days 0, 7, and 28. Kruskal–Wallis test was used for comparison. Bonferroni's correction was used, and adjusted *P* values were represented in this figure. Top of bars in (B) represents mean value, and top of bars in other plots represents median value. *p < .05. CHB, chronic hepatitis B virus infection; IgG, immunoglobulin G.

As shown in Figure 2A and Figure S4A, after influenza vaccination, ASC frequencies (defined as CD3^−^CD19^+^CD20^low/−^CD27^high^CD38^high^) in patients and HCs peaked on day 7 (7 days after influenza vaccination) (*p* < .001) and then decreased significantly on day 28 (*p* < .01). The frequencies of ASCs were significantly lower in CHB patients, especially in those with cirrhosis, than in HCs on day 28 (0.10 vs. 0.30, *p* = .014) (Figure 2A and Figure S4A). In general, the frequencies of H1N1‐specific IgG^+^ PBMCs appeared to increase after vaccination but without statistical significance (Figure S4B). No significant difference was found between the patients and HCs (Figure 2B and Figure S4B).

Overall, dynamics of the three cTFH cell subpopulations (cTFH1 [CD3^+^CD4^+^CXCR5^+^CXCR3^+^CCR6^−^], cTFH2 [CD3^+^CD4^+^CXCR5^+^CXCR3^−^CCR6^−^], and cTFH17 [CD3^+^CD4^+^CXCR5^+^CXCR3^−^CCR6^+^]) differed after influenza vaccination (Figure 2C–E and Figure S4C–E). On days 0 and 28, CHB patients (especially those with cirrhosis) had higher frequencies of cTFH1 cells (*p* < .05), similar frequencies of cTFH2 cells, and lower frequencies of cTFH17 cells (*p* < .05) than HCs. On day 7, the frequencies of cTFH1, 2 and 17 cells were similar in patients and HCs (Figure 2C–E and Figure S4C–E). We further analyzed ICOS^+^cTFH1 cells, the subpopulation that is closely associated with the antibody response after vaccination, and the dynamics were very similar to those of ASCs (Figure 2A and 2F and Figure S4A and S4F). Nevertheless, no difference in the frequencies of ICOS^+^cTFH1 cells was found between the patients and HCs on days 0, 7, and 28 (Figure 2F and Figure S4F).

### 3.4 Association between anti‐influenza antibody and clinical/immunological characteristics

To further explore the possible factors that impact the anti‐influenza antibody levels in CHB patients, we performed two‐variable and multivariate linear regression analyses; variables with *p* values less than .01 in two‐variable analyses were included in multivariate linear regression analyses. Anti‐H1N1 IgG levels on day 28 correlated positively with the frequencies of ASCs on day 7 (*r* = .620, *p* < .001) and negatively with HBsAg levels and liver cirrhosis (*p* < .05) (Table 2).

**Table 2:** Correlation between anti‐H1N1 IgG levels and clinical/immunological characteristics

	Two‐variable correlation analysis		Multiple linear regression	
	*r*	*p* value	*β* (95% CI)	*p* value
Age (years)	−.056	.634		
Gender (female)	.146	.126		
BMI (kg/m^2^)	**−.240**	**.038**		
RBC (10^9^/L)	−.057	.624		
PLT (10^9^/L)	**.257**	**.026**		
WBC (10^9^/L)	.209	.073		
Lymphocyte (10^9^/L)	.149	.203		
ALT (U/L)	−.072	.537		
AST (U/L)	−.143	.220		
TB (μmol/L)	−.135	.321		
HBsAg (IU/ml)	**−.267**	**.047**	**−.263**	**.007**
HBeAg‐positive	−.088	.427		
HBV DNA (IU/ml)	.145	.285		
Antiviral treatment	−.204	.066		
Liver cirrhosis	**−.220**	**.048**	**−.190**	**.043**
B cell day 0 (number/μl)	.023	.848		
B cell day 7 (number/μl)	.125	.287		
B cell day 28 (number/μl)	.001	.991		
CD4^+^ T cell day 0 (number/μl)	.184	.115		
CD4^+^ T cell day 7 (number/μl)	.123	.291		
CD4^+^ T cell day 28 (number/μl)	**.247**	**.033**		
ASC day 0 (number/μl)	.166	.155		
ASC day 7 (number/μl)	**.297**	**.010**	**.620**	**<.001**
ASC day 28 (number/μl)	.068	.563		
ICOS^+^ cTFH1 day 0 (number/μl)	.066	.575		
ICOS^+^ cTFH1 day 7 (number/μl)	.128	.274		
ICOS^+^ cTFH1 day 28 (number/μl)	−.065	.580		
cTFH1 day 0 (%)	.132	.259		
cTFH1 day 7 (%)	.127	.278		
cTFH1 day 28 (%)	.037	.753		
cTFH2 day 0 (%)	.131	.263		
cTFH2 day 7 (%)	.081	.492		
cTFH2 day 28 (%)	.189	.104		
cTFH17 day 0 (%)	−.129	.271		
cTFH17 day 7 (%)	−.192	.099		
cTFH17 day 28 (%)	−.135	.249		
H1N1‐specific IgG^+^ PBMCs day 0 (number)	−.097	.410		
H1N1‐specific IgG^+^ PBMCs day 7 (number)	.166	.166		
H1N1‐specific IgG^+^ PBMCs day 28 (number)	−.157	.179		
Anti‐H1N1 IgG day 0 (μg/ml)	**.301**	**.009**		

*Note*: Spearman's rank correlation (for two continuous variables) and Kendall's rank correlation (when one of the variables was discontinuous) were used for two‐variable correlation analysis. When the *p* value of a variable is lower than 0.1 (**bold**), this variable was included in multivariate linear regression analysis (step method) for the further analysis.

Abbreviations: ALT, alanine aminotransferase; ASC, antibody‐secreting cell; AST, aspartate aminotransferase; BMI, body mass index; CI, confidential interval; cTFH, circulating T follicular helper; ICOS, inducible costimulatory; PBMCs, peripheral blood mononuclear cells. PLT, platelet; RBC, red blood cell; TB, total bilirubin; WBC, white blood cell.

### 3.5 Relationship between influenza vaccination and anti‐SARS‐CoV‐2 antibody

During 1‐year follow‐up after influenza vaccination, 8 CHB patients were subsequently fully vaccinated against COVID‐19 (group 1). We propensity matched 64 CHB patients and 46 HCs who only received two doses of inactivated SARS‐CoV‐2 vaccine as the controls (groups 2 and 3, respectively). Detailed characteristics of these persons were shown in Table S4–6.

**Figure 3:**
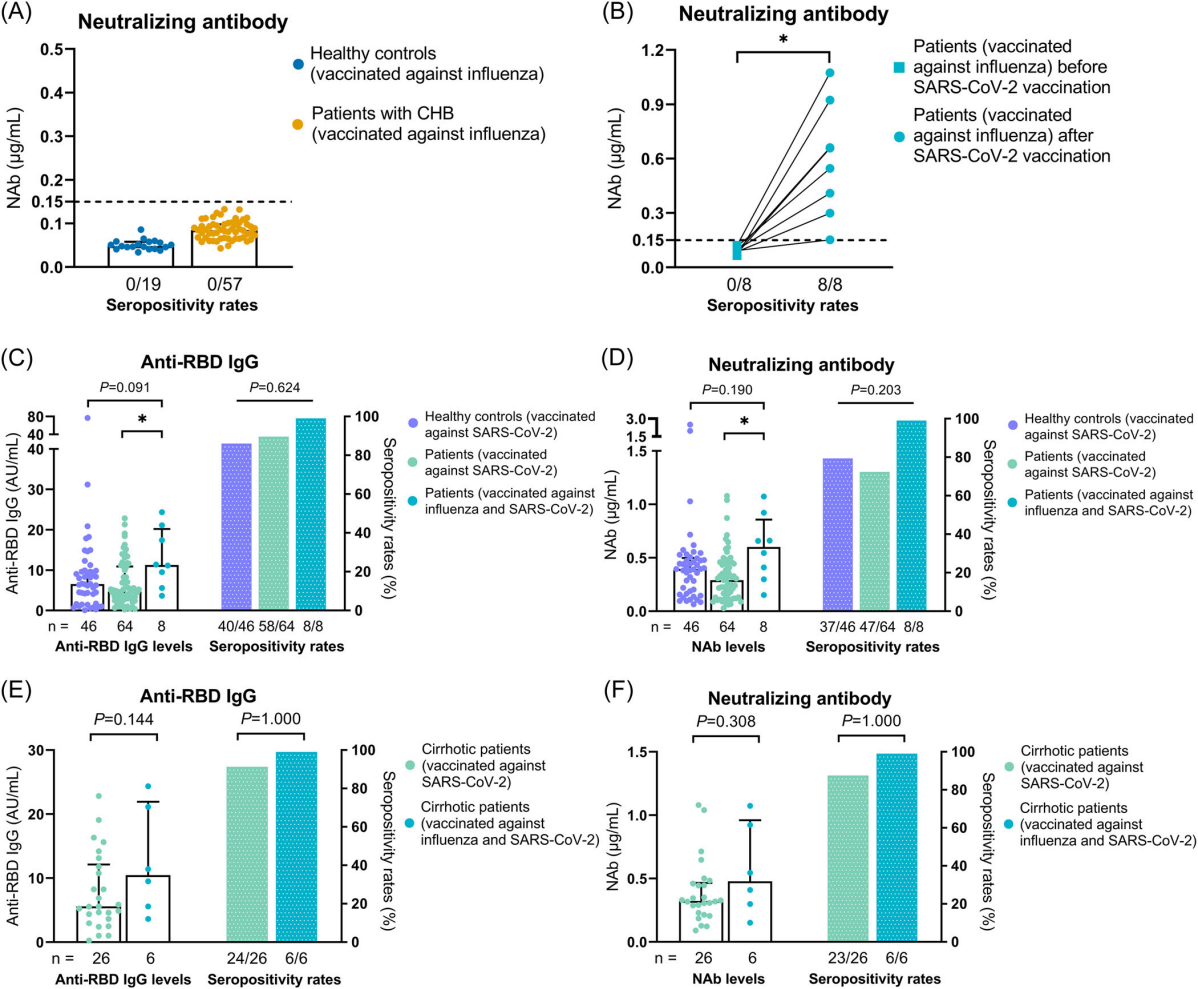
Antibody response to SARS‐CoV‐2 vaccine. (A) NAb levels in healthy controls and patients with CHB at 28 days after the influenza vaccination. (B) NAb levels before and after the two‐dose inactivated SARS‐CoV‐2 vaccination in the 8 patients with CHB who had a history of influenza vaccination within 1 year. (C, D) Anti‐RBD IgG (C) and NAb (D) levels in the 46 healthy controls and 64 patients with CHB who only completed two‐dose inactivated SARS‐CoV‐2 vaccination and the 8 patients with CHB who completed two‐dose inactivated SARS‐CoV‐2 vaccination within 1 year after influenza vaccination. (E, F) Anti‐RBD IgG (E) and NAb (F) levels in the 26 cirrhotic patients with CHB who only completed two‐dose inactivated SARS‐CoV‐2 vaccination and the 6 cirrhotic patients with CHB who completed two‐dose inactivated SARS‐CoV‐2 vaccination within 1 year after influenza vaccination. Wilcoxon signed‐rank test was used in (B). Dunn's multiple comparisons test was used in (C, D), and *p* values represented in this figure are adjusted *p* values. Chi‐Square and Fisher's exact tests were used in (C–F). Mann–Whitney *U* test were used in (E, F). Top of all bars represents median value, and error bars represent interquartile range. *p < .05.

A strong positive correlation existed between anti‐RBD IgG and NAb (*r* = .879, *p* < .001) (Figure S5). The patients of group 1 were tested negative for NAb before SARS‐CoV‐2 vaccination, and positive at 1 month after full vaccination (Figure 3B).

Anti‐RBD IgG (100% vs. 90.6% vs. 87.0%, *p* = .624) and NAb (100% vs. 73.4% vs. 80.4%, *p* = .203) seropositivity rates were similar among the three groups. But anti‐SARS‐CoV‐2 antibody levels appeared to be higher in group 1 than in other groups, especially group 2 (anti‐RBD IgG: 11.28 vs. 4.67, *p* = .029; NAb: 0.602 vs. 0.290, *p* = .019) (Figure 3C,D). Furthermore, anti‐RBD IgG (10.45 vs. 5.59, *p* = .144) and NAb (0.478 vs. 0.324, *p* = .308) levels were similar between the group 1 patients with cirrhosis and the group 2 patients with cirrhosis (Figure 3E,F).

In all three models of multivariate linear regression analyses, a history of influenza vaccination within 1 year before two‐dose inactivated SARS‐CoV‐2 vaccination correlated positively with anti‐RBD IgG and NAb levels (*p* < .01) (Table 3). We also combined two‐variable and multivariate linear regression analyses and included variables with *p* values less than .01 in the two‐variable analyses in the multivariate linear regression analyses. These results also showed that a history of influenza vaccination within 1 year correlated with higher anti‐RBD IgG and NAb levels (*p* < .05) (Table S7).

**Table 3:** Association between influenza vaccination and anti‐SARS‐CoV‐2 antibody levels

	Model 1				Model 2				Model 3			
	Anti‐RBD IgG		NAb		Anti‐RBD IgG		NAb		Anti‐RBD IgG		NAb	
	***β***	***p***	***β***	***p***	***β***	***p***	***β***	***p***	***β***	***p***	***β***	***p***
Influenza vaccination												
No												
Yes	.367	.002	.393	.001	.345	.005	.351	.004	.344	.005	.349	.004

*Note*: Multivariate linear regression analysis was used. Model 1 included age, gender, body mass index, inactivated SARS‐CoV‐2 vaccines, and interval days between SARS‐CoV‐2 vaccination and blood sampling for adjustment. Model 2 included variables in Model 1 and liver cirrhosis for adjustment. Model 3 included variables in Model 2 and hepatitis B surface antigen for adjustment. β represents the standardized regression coefficient.

Abbreviations: NAb, neutralizing antibody; RBD, receptor binding domain.

## 4 DISCUSSION

This study focused on comparison of the humoral immune response between patients with CHB and HCs after influenza vaccination and identified influencing factors of the anti‐influenza antibody response using multivariate analysis. Furthermore, through comparison between groups and multivariate analysis, we explored the impact of a history of influenza vaccination within 1 year before inactivated SARS‐CoV‐2 vaccination on the anti‐SARS‐CoV‐2 antibody response to the inactivated SARS‐CoV‐2 vaccine.

Our previous study revealed good safety of inactivated SARS‐CoV‐2 vaccines in patients with CHB.^12^ Similarly, in the present study, no SAEs occurred in patients with CHB after influenza vaccination, and no severe increase/decrease in levels of routine blood test parameters, liver function indices or HBV DNA was observed. These results suggest that inactivated influenza vaccines are also safe in patients with CHB.

At 28 days after influenza vaccination, levels of three influenza antibodies and seropositivity rates seemed to be lower in non‐cirrhotic patients with CHB than in HCs, but the difference was not statistically significant. This indicates that CHB might slightly attenuate the antibody response to an inactivated influenza vaccine, which is similar to our previous study on patients with CHB vaccinated against SARS‐CoV‐2.^12^ Some studies have shown that liver cirrhosis^18^, ^19^ and HBsAg^20^ may inhibit the immune response and that liver cirrhosis may reduce the antibody response to influenza vaccines.^21^ In this study, CHB patients with cirrhosis appeared to have lower levels of the three influenza antibodies on day 28 than HCs, and the difference in anti‐H1N1 IgG was statistically significant. Furthermore, multivariate analyses showed that liver cirrhosis and HBsAg levels correlated significantly negatively with anti‐H1N1 IgG levels. These results indicated that patients with CHB who have cirrhosis/high HBsAg levels may exhibit a lower antibody response to influenza vaccines.

To explore the mechanism of the antibody response, we analyzed ASCs, cTFH cells, and H1N1‐specific IgG PBMCs. ASCs are a type of B cell that secretes antibodies,^22^ and previous studies showed that the ASC frequencies on day 7 were positively correlated with anti‐influenza antibody levels on day 28.^23^, ^24^ Similarly, this study found that ASC frequencies on day 7 correlated positively with anti‐H1N1 IgG levels on day 28. Though ASC frequencies on day 28 were significantly lower in cirrhotic patients than in HCs, no correlation was found between ASC frequencies and anti‐H1N1 IgG levels on day 28. The results indicated that the difference in the ASC frequencies on day 28 may not be the cause of the difference in anti‐H1N1 IgG levels. cTFH cells comprise subpopulations of CD4^+^ T cells that provide help to B cells and induce the antibody response.^25^ However, in this study, frequencies of cTFH1, −2, and −17 cells did not correlate significantly with anti‐influenza antibody levels. As ICOS^+^ cTFH1 cells are reportedly closely related to antibody production after inactivated influenza vaccination,^26^ we further analyzed the relationship between ICOS^+^cTFH1 cells and influenza antibody levels. However, we found no significant difference in ICOS^+^cTFH1 cell frequencies between patients and HCs, indicating that the difference in antibody levels between patients and HCs may not be due to ICOS^+^cTFH1 cells.

Some studies have revealed that Bacille Calmette‐Guérin and tetanus‐diphtheria‐pertussis vaccinations may affect the immune response to subsequent vaccines/pathogens.^27^, ^28^, ^29^ Similarly, a recent study reported that influenza vaccination affects the cytokine response to SARS‐CoV‐2.^13^ In our study, neither patients with CHB nor HCs had developed NAbs against SARS‐CoV‐2 by 28 days after inactivated influenza vaccination. Nonetheless, interestingly, after two‐dose inactivated SARS‐CoV‐2 vaccination, anti‐SARS‐CoV‐2 antibody levels were significantly higher in patients with a history of influenza vaccination within 1 year than in patients without a history. Furthermore, multivariate analyses demonstrated positive correlations between an influenza vaccination history and anti‐SARS‐CoV‐2 antibody levels. Therefore, the results suggest that influenza vaccination might affect the immune response to the inactivated SARS‐CoV‐2 vaccine and might partially explain why influenza vaccination reduces SARS‐CoV‐2 infection, severe symptoms, and death.^14^, ^15^, ^16^ In this study, influenza vaccination increased the antibody response to the inactivated SARS‐CoV‐2 vaccine, which might be caused by trained immunity,^30^ but the specific mechanism needs to be further studied.

This study has several advantages. First, this study explored whether CHB and liver cirrhosis affect the immune response to vaccines and preliminarily explored the related mechanisms. Second, this study investigated the impact of influenza vaccination on the antibody response to the inactivated SARS‐CoV‐2 vaccine, and the results partially explain why influenza vaccination improves COVID‐19‐related outcomes. However, there are also several limitations. First, although influenza vaccination was found to may enhance the antibody response to the inactivated SARS‐CoV‐2 vaccine, further research is needed to reveal the mechanism. Second, globally, the currently widely used SARS‐CoV‐2 vaccines include inactivated, mRNA and viral vector vaccines, all of which can induce effective antibody responses against SARS‐CoV‐2.^31^ However, since mRNA and viral vector vaccines were unavailable in China mainland, all participants in our study were only vaccinated with inactivated vaccines. Third, the number of participants in this study is relatively low, and studies with a large number of participants are needed to verify our findings in the future.

In conclusion, this study shows that the antibody response to an inactivated influenza vaccine appears to be reduced in patients with CHB, especially in those with cirrhosis. A history of inactivated influenza vaccination within 1 year before inactivated SARS‐CoV‐2 vaccination might result in a stronger anti‐SARS‐CoV‐2 antibody response.

## AUTHOR CONTRIBUTIONS

*Concept and design*: Hong Ren, Min Chen, Taiyu He. *Funding acquisition*: Hong Ren, Peng Hu, Min Chen. *Participant recruitment*: Ning Ling, Taiyu He, Peng Hu, Dachuan Cai, Dazhi Zhang. *Experiment execution*: Taiyu He, Gaoli Zhang, Dejuan Xiang, Min Chen, Mingli Peng. *Data analysis*: Taiyu He, Gaoli Zhang. *Drafting and critical revision of manuscript*: Taiyu He, Ning Ling, Gaoli Zhang, Dejuan Xiang, Peng Hu, Mingli Peng, Dachuan Cai, Dazhi Zhang, Hong Ren, Min Chen. All authors contributed to the article and approved the submitted version.

## CONFLICTS OF INTERESTS

The authors declare no conflicts of interests.

## ETHICS STATEMENT

This study was approved by the ethics committee of the Second Affiliated Hospital of Chongqing Medical University and registered at ClinicalTrials.gov (NCT05007665, date: August 16, 2021) and conformed to the ethical guidelines of the Declaration of Helsinki.

## Supporting information

Supporting information.

## Data Availability

The authors declare that all data included in this article will be made available upon reasonable request.
