# Supplementary material for: Decreased antibody response to influenza vaccine with an enhanced antibody response to subsequent SARS‐CoV‐2 vaccination in patients with chronic hepatitis B virus infection
Source: Immun Inflamm Dis. 2022 Dec 31;11(1):e759. doi: 10.1002/iid3.759 (PMC9803931; doi:10.1002/iid3.759)
Supplement: Supplementary file 1 — Supporting information. [file IID3-11-0-s001.docx]

Decreased antibody response to influenza vaccine with an enhanced antibody response to subsequent SARS-CoV-2 vaccination in patients with chronic hepatitis B virus infection

Taiyu He^#^, Ning Ling^#^, Gaoli Zhang^#^, Dejuan Xiang, Peng Hu, Mingli Peng, Dachuan Cai, Dazhi Zhang, Min Chen^*^, Hong Ren^*^

Table of contents

Supplementary results.......................................................................................2

Figure S1...........................................................................................................2

Figure S2...........................................................................................................3

Figure S3...........................................................................................................4

Figure S4...........................................................................................................5

Figure S5...........................................................................................................6

Table S1............................................................................................................7

Table S2............................................................................................................8

Table S3............................................................................................................9

Table S4..........................................................................................................10

Table S5..........................................................................................................11

Table S6..........................................................................................................12

Table S7..........................................................................................................13


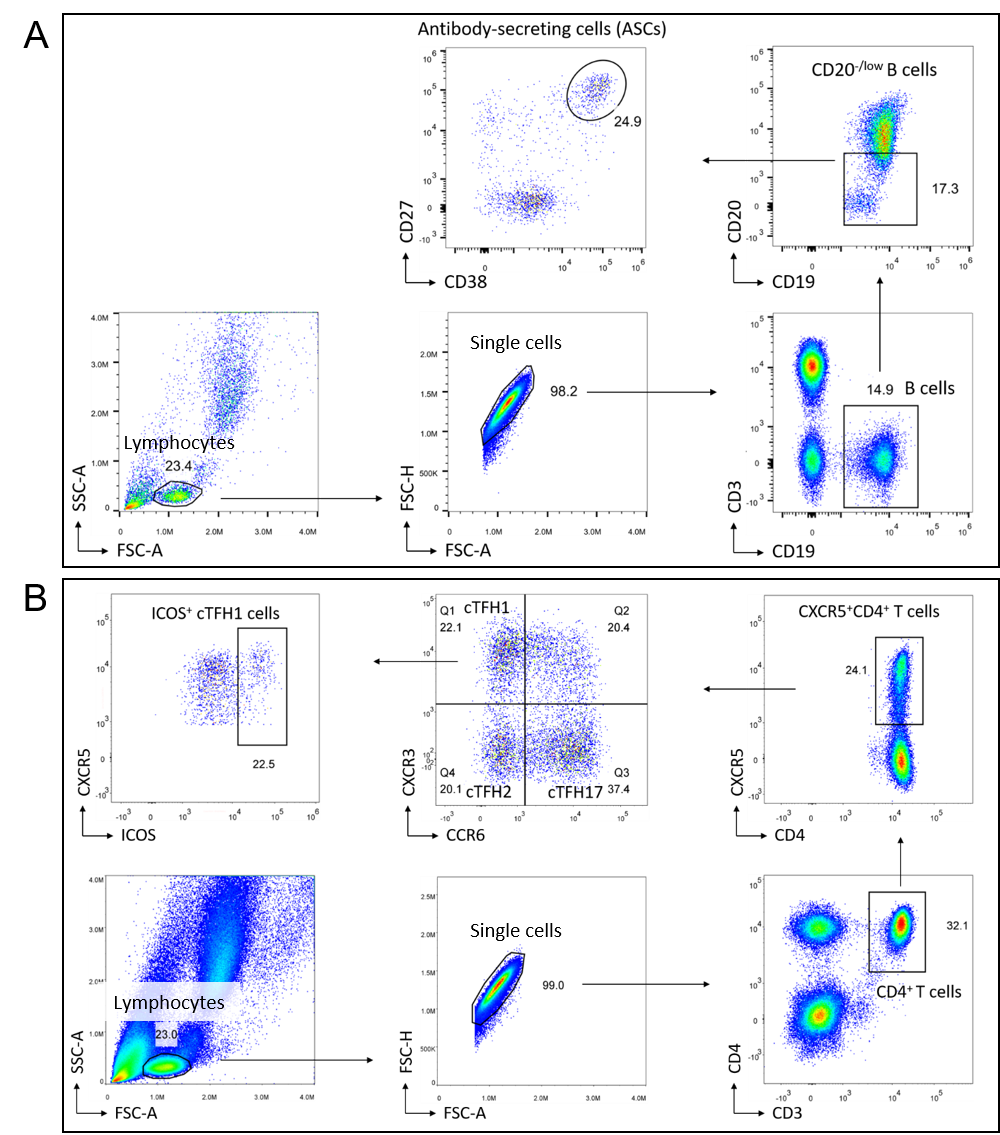


**Figure S1. Gating strategies for ASCs and cTFH cells. (A, B)** Gating strategies for ASCs **(A)** and cTFH cells **(B)** in flow cytometry. ASCs, antibody-secreting cells; cTFH, circulating T follicular helper.

**
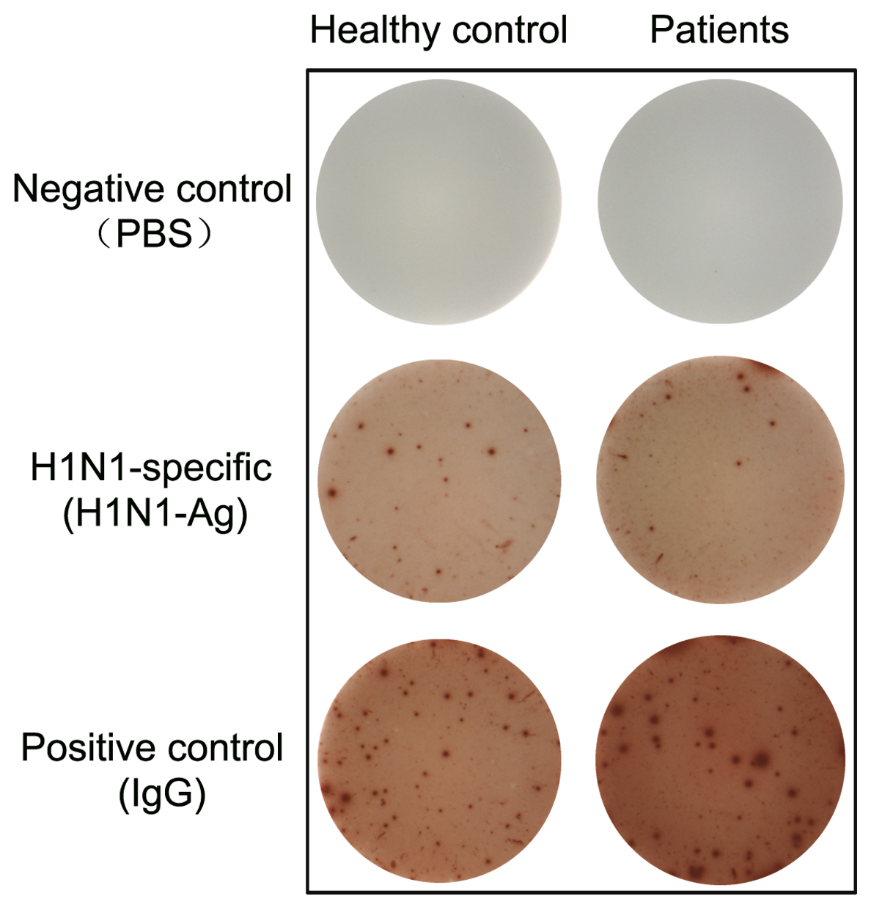
**

**Figure S2. ELISPOT assay image for H1N1-specific IgG^+^ PBMCs.** The dots in the figure represent IgG^+^ PBMCs. PBMCs, peripheral blood mononuclear cells.


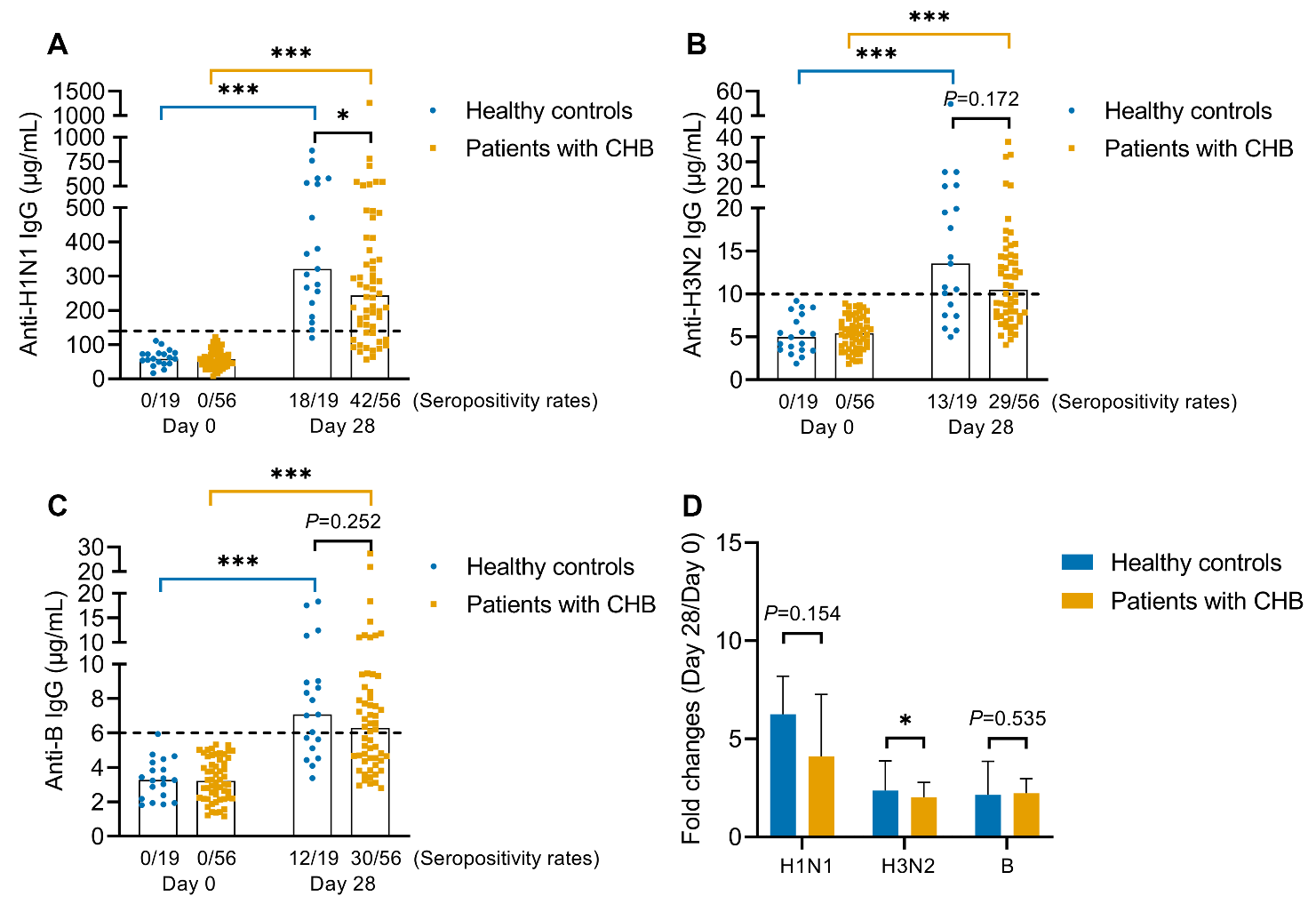
**Figure S3. Antibody response to influenza vaccine.** **(A-C)** Anti-H1N1 IgG **(A)**, anti-H3N2 IgG **(B)**, and anti-B IgG **(C)** levels in HCs and all patients with CHB at day 0 and 28. **(D)** Fold changes of antibody levels (day 28/day 0) in HCs and all patients with CHB. Wilcoxon signed-rank test and Mann-Whitney U test were used for comparison. Top of all bars represents median value. **P* < 0.05, ****P* < 0.001. Day 0 represents the influenza vaccination day, day 7 represents 7 days after influenza vaccination, and day 28 represents 28 days after influenza vaccination. HCs, healthy controls.


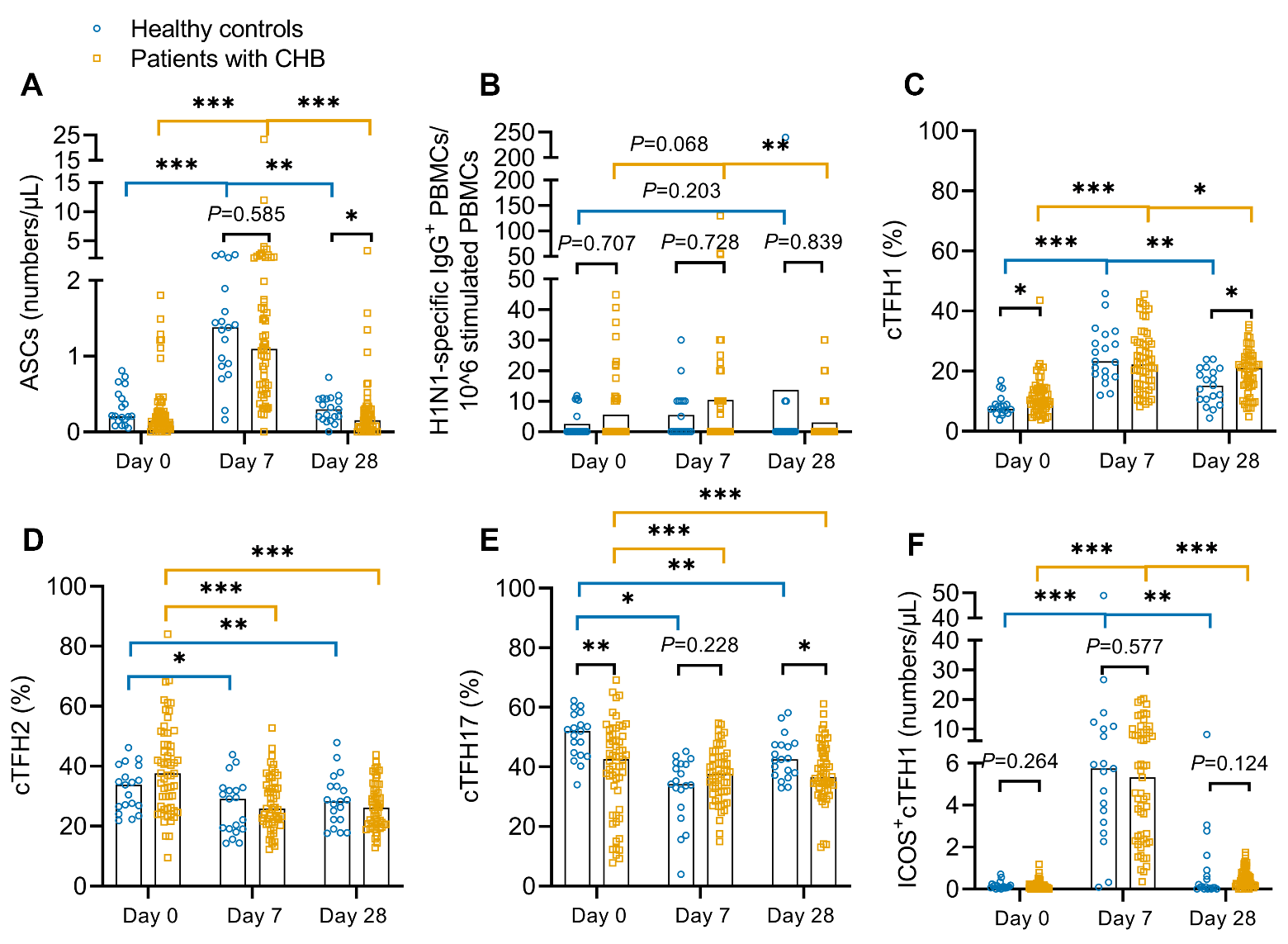
**Figure S4. Cell response to influenza vaccine in healthy controls and patients with CHB.** **(A-F)** Frequencies of ASCs **(A)**, H1N1-specific IgG^+^ PBMCs **(B)**, cTFH1 **(C)**, cTFH2 **(D)**, cTFH17 **(E)** and ICOS^+^cTFH1 **(F)** cells in healthy controls and all patients with CHB at day 0, 7 and 28. Mann-Whitney U test and Friedman test were used for comparison. Bonferroni's correction was used, and adjusted *P* values were represented in this figure. Top of bars in **(B)** represents mean value, and top of bars in other plots represents median value. **P* < 0.05, ***P* < 0.01, ****P* < 0.001. ASCs, antibody-secreting cells; cTFH, circulating T follicular helper; PBMCs, peripheral blood mononuclear cells.


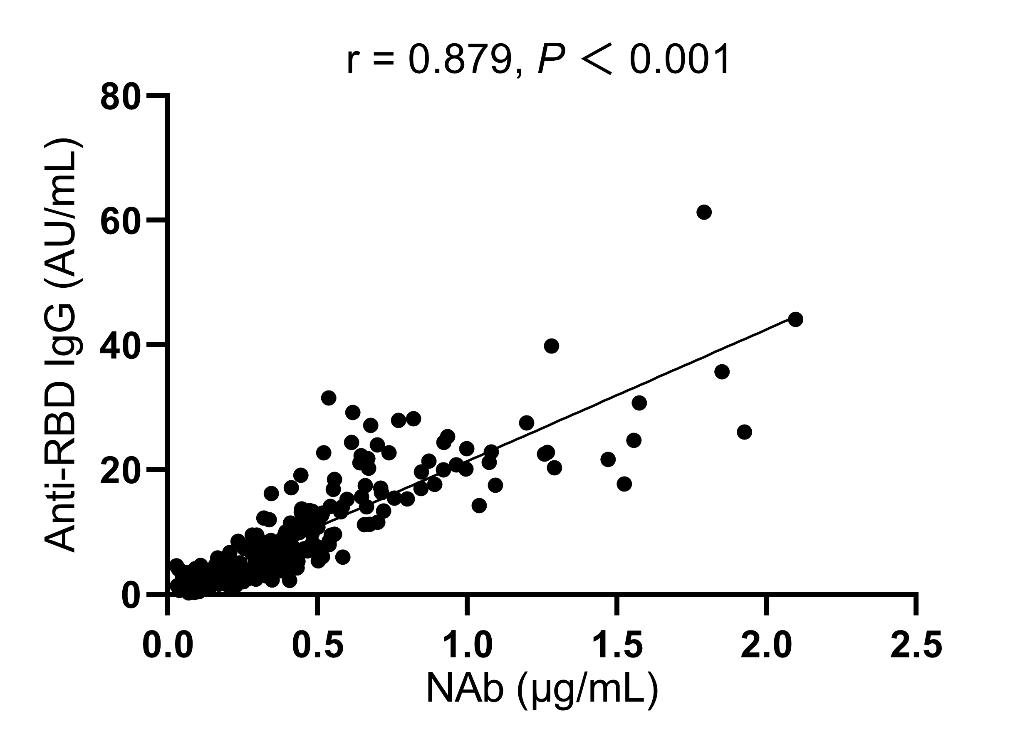


**Figure S5. Correlation between anti-RBD IgG and NAb.** Spearman’s rank correlation was used, and r represents correlation coefficient.

| **Table S1. Characteristics of healthy controls, non-cirrhotic and cirrhotic patients who got flu vaccination** | | | | |
| --- | --- | --- | --- | --- |
| **Variables** | **Healthy controls**  **(n=19)** | **Patients without cirrhosis**  **(n=35)** | **Patients with cirrhosis**  **(n=22)** | ***P* value** |
| Age^#^ (years) | 51(24-62) | 49 (25-65) | 53 (29-67) | 0.886 |
| 18-60, n (%) | 17 (89.5%) | 28 (80.0%) | 17 (77.3%) | 0.646 |
| ≥60, n (%) | 2 (10.5%) | 7 (20.0%) | 5 (22.7%) |  |
| Gender |  |  |  |  |
| Male, n (%) | 9 (47.4%) | 18 (51.4%) | 14 (63.6%) | 0.535 |
| Female, n (%) | 10 (52.6%) | 17 (48.6%) | 8 (36.4%) |  |
| BMI^#^ (kg/m^2) | 23.69 (19.05-28.08) | 23.88 (18.99-29.28) | 24.36 (18.52-31.57) | 0.571 |
| RBC^#^ (10^9/L) | 4.48 (3.98-5.77) | 4.60 (4.04-5.76) | 4.52 (3.68-5.64) | 0.481 |
| PLT^#^ (10^9/L) | 187 (78-321) | 189 (114-302) | 119 (39-261) | **＜0.001** |
| WBC^#^ (10^9/L) | 6.86 (3.64-10.55) | 6.17 (3.61-12.14) | 5.57 (1.38-7.79) | 0.059 |
| Lymphocyte^#^ (10^9/L) | 1.98 (1.15-3.26) | 1.92 (0.90-3.00) | 1.51 (0.28-2.60) | 0.069 |
| ALT^#^ (U/L) | 19 (9-49) | 23 (8-95) | 26 (8-84) | 0.105 |
| AST^#^ (U/L) | 20 (13-38) | 23 (15-66) | 32 (16-76) | **＜0.001** |
| HBsAg |  |  |  |  |
| ＜250 IU/mL, n (%) | / | 21 (60.0%) | 11 (50.0%) | 0.459 |
| ≥250 IU/mL, n (%) | / | 14 (40.0%) | 11 (50.0%) |  |
| HBeAg (positive, n (%)) | / | 10 (28.6%) | 2 (9.1%) | 0.155 |
| HBV DNA^#^ (IU/mL) | / | 100 (100-1.75×10^8) | 100 (20-100) | **＜0.001** |
| Antiviral treatment, n (%) | / | 19 (54.3%) | 22 (100%) | **＜0.001** |
| ^#^Presented as median (range). The Chi-Square test and Fisher’s exact test were used for categorical variables. Mann-Whitney U test and Kruskal-Wallis test were used for continuous variables. The lower limitation of HBV DNA detection is 20 IU/mL. When the HBV DNA levels were lower than 20 IU/mL, a 20 IU/mL was assigned. | | | | |
| ALT, alanine aminotransferase; AST, aspartate aminotransferase; BMI, body mass index; HBeAg, hepatitis B e antigen; PLT, platelet; RBC, red blood cell; WBC, white blood cell | | | | |

| **Table S2.** Adverse events within 28 days after influenza vaccination | | | |
| --- | --- | --- | --- |
|  | **Healthy controls (n=19)** | **Patients (n=57)** | ***P* value** |
| **Overall adverse events within 28 days** | 2 (10.5%) | 5 (8.6%) | 1.000 |
| **Grade 3 and 4 adverse events** | / | / | / |
| **Local adverse events** |  |  |  |
| Pain | / | 2 (3.5%) | 1.000 |
| Swelling | 1 (5.3%) | 1 (1.8%) | 1.000 |
| Redness | / | / | / |
| **Systemic adverse events** |  |  |  |
| Fatigue | 1 (5.3%) | 1 (1.8%) | 1.000 |
| Cough | / | 1 (1.8%) | 1.000 |
| Diarrhea | / | / | / |
| Fever | / | / | / |
| Headache | / | / | / |
| Muscle pain | / | / | / |
| Nausea | / | / | / |
| Data are presented as n (%). Chi-square test and Fisher’s exact test were used to compare statistical difference between groups. | | | |

| **Table S3. Laboratory parameters before and after influenza vaccination in patients** | | | | |
| --- | --- | --- | --- | --- |
| **Variables** | **Day 0** | **Day 7** | **Day 28** | ***P* value** |
| RBC^#^ (10^9/L) | 4.57 (3.68-5.76) | 4.75 (3.72-5.94) | 4.72 (3.82-5.75) | **0.000** |
| PLT^#^ (10^9/L) | 157 (39-302) | 158 (36-299) | 150 (38-330) | 0.178 |
| WBC^#^ (10^9/L) | 5.84 (1.38-12.14) | 5.58 (1.54-9.46） | 5.38 (2.48-10.40) | **0.001** |
| Lymphocyte^#^ (10^9/L) | 1.75 (0.28-3.00) | 1.91 (0.30-3.22） | 1.68 (0.50-3.26) | **0.001** |
| ALT^#^ (U/L) | 25 (8-95) | 25 (11-122) |  | 0.573 |
| AST^#^ (U/L) | 25 (15-76) | 26 (15-65) |  | 0.603 |
| TB^#^ (μmol/L) | 9.5 (4.2-44) | 10.6 (3.9-27.2) |  | 0.535 |
| DB^#^ (μmol/L) | 3.8 (2-16.7) | 3.8 (1.9-11.2) |  | 0.053 |
| IB^#^ (μmol/L) | 6.0 (1.1-27.3) | 7.1 (1.8-18.6) |  | 0.136 |
| ALP^#^ (U/L) | 83 (46-170) | 84 (45-198) |  | **0.006** |
| GGT^#^ (U/L) | 31 (10-138) | 30 (10-121) |  | **0.002** |
| TBA^#^ (μmol/L) | 9.0 (2.4-171.6) | 5.0 (1.0-71.9） |  | **0.001** |
| PAB^#^ (μmol/L) | 205 (57-369) | 211 (94-334) |  | 0.112 |
| CHE^#^ (KU/L) | 8.86 (3.38-24.68) | 9.23 (3.49-20.87) |  | 0.446 |
| HBV DNA^#^ (IU/mL) | 100 (20-1.75×10^8) |  | 100 (20-6.47×10^8) | 0.408 |
| ^#^Presented as median (range). Wilcoxon signed-rank test and Friedman test were used for comparison.  ALP, alkaline phosphatase; ALT, alanine aminotransferase; AST, aspartate aminotransferase; CHE, cholinesterase; DB, direct bilirubin; GGT, gamma-glutamyltranspeptidase; IB, indirect bilirubin; PAB, prealbumin; PLT, platelet; RBC, red blood cell; TB, total bilirubin; TBA, total bile acid; WBC, white blood cell. | | | | |

| **Table S4. Characteristics of the 8 patients with CHB vaccinated against influenza and subsequently SARS-CoV-2** | | | | | | | | |
| --- | --- | --- | --- | --- | --- | --- | --- | --- |
| **Variables** | Patient 1 | Patient 2 | Patient 3 | Patient 4 | Patient 5 | Patient 6 | Patient 7 | Patient 8 |
| Age (years) | 30 | 57 | 58 | 34 | 62 | 64 | 57 | 58 |
| Gender | Male | Male | Male | Male | Female | Female | Female | Female |
| BMI (kg/m^2) | 22.0 | 25.9 | 24.2 | 27.3 | 25.4 | 26.7 | 22.4 | 24.7 |
| RBC (10^9/L) | 5.61 | 5.20 | 4.98 | 5.60 | 3.95 | 5.27 | 4.77 | 4.64 |
| PLT (10^9/L) | 212 | 136 | 181 | 194 | 237 | 132 | 156 | 92 |
| WBC (10^9/L) | 5.28 | 6.70 | 7.33 | 8.71 | 5.71 | 5.76 | 5.97 | 4.55 |
| Lymphocyte (10^9/L) | 2.44 | 1.61 | 2.07 | 1.66 | 2.18 | 2.18 | 1.26 | 1.47 |
| ALT (U/L) | 64 | 23 | 40 | 29 | 23 | 17 | 11 | 21 |
| AST (U/L) | 39 | 26 | 31 | 26 | 32 | 29 | 19 | 40 |
| TB (μmol/L) | 11.9 | 10.7 | 14.3 | 31.3 | 14.3 | 6.9 | 11.2 | 28.9 |
| HBsAg (IU/mL) | 250.00 | 42.86 | 4560.95 | 250.00 | 26.22 | 225.52 | 250.00 | 172.81 |
| HBeAg | Positive | Negative | Negative | Negative | Negative | Negative | Negative | Negative |
| HBV DNA (IU/mL) | 100 | 20 | 1530 | 100 | 20 | 20 | 20 | 20 |
| Antiviral treatment | ETV | ETV | No | ETV | ETV | ETV | TDF | ETV |
| Liver cirrhosis | Yes | Yes | No | Yes | Yes | Yes | No | Yes |
| Anti-H1N1 IgG day 28 (μg/mL) | 294.0 | 152.7 | 411.6 | 471.0 | 707.7 | 275.3 | 285.5 | 268.2 |
| Anti-H3N2 IgG day 28 (μg/mL) | 7.8 | 32.2 | 5.1 | 11.9 | 13.0 | 12.0 | 6.2 | 13.7 |
| Anti-B IgG day 28 (μg/mL) | 8.4 | 7.2 | 7.1 | 4.7 | 7.6 | 3.8 | 5.1 | 9.3 |
| Days between the Influenza vaccination and second inactivated SARS-CoV-2 vaccination | 203 | 209 | 191 | 286 | 285 | 290 | 291 | 314 |
| Inactivated vaccine | CoronaVac | BBIBP-CorV | CoronaVac | BBIBP-CorV | BBIBP-CorV | CoronaVac | CoronaVac | CoronaVac |
| Days between the second inactivated SARS-CoV-2 vaccination and blood sampling | 28 | 30 | 40 | 21 | 25 | 22 | 28 | 22 |
| Anti-RBD IgG (AU/mL) | 21.14 | 3.64 | 11.16 | 5.60 | 9.51 | 11.40 | 17.42 | 24.37 |
| NAb (μg/mL) | 1.074 | 0.152 | 0.658 | 0.299 | 0.546 | 0.410 | 0.661 | 0.923 |
| Notes: The upper limitation of semi-quantitative method for detecting HBsAg levels is 250 IU/mL, and the semi-quantitative method was used in patient 1, 4 and 7. Their HBsAg levels were detected higher than 250 IU/mL, so a 250 IU/mL was assigned. The lower limitation of HBV DNA detection is 20 IU/mL. When the HBV DNA levels were lower than 20 IU/mL, a 20 IU/mL was assigned.  ALT, alanine aminotransferase; AST, aspartate aminotransferase; BMI, body mass index; ETV, entecavir; PLT, platelet; RBC, red blood cell; TB, total bilirubin; TDF, tenofovir disoproxil fumarate; WBC, white blood cell. | | | | | | | | |

| **Table S5. Characteristics of patients with CHB vaccinated with inactivated SARS-CoV-2 vaccine** | | | |
| --- | --- | --- | --- |
| **Variables** | **Patients only vaccinated against SARS-CoV-2 (n=64)** | **Patients vaccinated against influenza and SARS-CoV-2 (n=8)** | ***P* value** |
| Age^#^ (years) | 50.5 (30-78) | 57.5 (30-64) | 0.070 |
| Gender |  |  |  |
| Male, n (%) | 32 (50.0%) | 4 (50.0%) | 1.000 |
| Female, n (%) | 32 (50.0%) | 4 (50.0%) |  |
| BMI^#^ (kg/m^2) | 23.8 (18.4-30.2) | 25.1 (22.0-27.3) | 0.324 |
| RBC^#^ (10^9/L) | 4.62 (3.82-7.20) | 5.09 (3.95-5.61) | 0.128 |
| PLT^#^ (10^9/L) | 164.0 (24-291) | 168.5 (92-237) | 0.628 |
| WBC^#^ (10^9/L) | 5.34 (1.95-7.95) | 5.87 (4.55-8.71) | 0.109 |
| Lymphocyte^#^ (10^9/L) | 1.81 (0.28-3.00) | 1.87 (1.26-2.44) | 0.720 |
| ALT^#^ (U/L) | 23.0 (10-756) | 23.0 (11-64) | 0.851 |
| AST^#^ (U/L) | 26.0 (12-627) | 30.0 (19-40) | 0.196 |
| TB^#^ (μmol/L) | 12.5 (4.3-54.9) | 13.1 (6.9-31.3) | 0.496 |
| HBsAg |  |  |  |
| ＜250 IU/mL, n (%) | 20 (31.3%) | 4 (50.0%) | 0.507 |
| ≥250 IU/mL, n (%) | 44 (68.7%) | 4 (50.0%) |  |
| HBeAg (positive, n (%)) | 20 (31.3%) | 1 (12.5%) | 0.492 |
| HBV DNA^#^ (IU/mL) | 20 (20-4.92×10^7) | 20 (20-1530) | 0.102 |
| Antiviral treatment, n (%) | 44 (68.8%) | 7 (87.5%) | 0.492 |
| Liver cirrhosis, n (%) | 26 (40.6%) | 6 (75.0%) | 0.142 |
| Inactivated vaccine |  |  |  |
| BBIBP-CorV n, (%) | 31 (48.4%) | 3 (37.5%) | 0.311 |
| CoronaVac n, (%) | 22 (34.4%) | 5 (62.5%) |  |
| BBIBP-CorV + CoronaVac n, (%) | 11 (17.2%) | 0 (0.0%) |  |
| Days between the second inactivated SARS-CoV-2 vaccination and blood sampling | 30.0 (21-42) | 26.5 (21-40) | 0.139 |
| Days between the Influenza vaccination and second inactivated SARS-CoV-2 vaccination | **/** | 285.5 (191-314) | **/** |
| ^#^Presented as median (range). The Chi-Square statistic and Fisher’s exact test were used for categorical variables, and Mann-Whitney U test was used for continuous variables.  ALT, alanine aminotransferase; AST, aspartate aminotransferase; BMI, body mass index; PLT, platelet; RBC, red blood cell; TB, total bilirubin; WBC, white blood cell | | | |

| **Table S6. Characteristics of HCs and patients vaccinated with inactivated SARS-CoV-2 vaccine** | | | |
| --- | --- | --- | --- |
| **Variables** | **HCs only vaccinated against SARS-CoV-2 (n=46)** | **Patients vaccinated against influenza and SARS-CoV-2 (n=8)** | ***P* value** |
| Age^#^ (years) | 44.0 (22-68) | 57.5 (30-64) | 0.243 |
| Gender |  |  |  |
| Male, n (%) | 18 (39.1%) | 4 (50.0%) | 0.851 |
| Female, n (%) | 28 (60.9%) | 4 (50.0%) |  |
| BMI^#^ (kg/m^2) | 23.8 (16.8-29.7) | 25.1 (22.0-27.3) | 0.172 |
| RBC^#^ (10^9/L) | 4.59 (3.65-6.68) | 5.09 (3.95-5.61) | 0.172 |
| PLT^#^ (10^9/L) | 196.0 (84-358) | 168.5 (92-237) | 0.092 |
| WBC^#^ (10^9/L) | 5.24 (2.09-11.32) | 5.87 (4.55-8.71) | 0.149 |
| Lymphocyte^#^ (10^9/L) | 1.73 (1.02-2.57) | 1.87 (1.26-2.44) | 0.566 |
| ALT^#^ (U/L) | 20.0 (4-48) | 23.0 (11-64) | 0.550 |
| AST^#^ (U/L) | 22.0 (12-35) | 30.0 (19-40) | **0.003** |
| TB^#^ (μmol/L) | 11.9 (5.1-29.5) | 13.1 (6.9-31.3) | 0.492 |
| HBsAg |  |  |  |
| ＜250 IU/mL, n (%) |  | 4 (50.0%) | / |
| ≥250 IU/mL, n (%) |  | 4 (50.0%) |  |
| HBeAg (positive, n (%)) |  | 1 (12.5%) | / |
| HBV DNA^#^ (IU/mL) |  | 20 (20-1530) | / |
| Antiviral treatment, n (%) |  | 7 (87.5%) | / |
| Liver cirrhosis, n (%) |  | 6 (75.0%) | / |
| Inactivated vaccine |  |  |  |
| BBIBP-CorV n, (%) | 18 (39.1%) | 3 (37.5%) | 1.000 |
| CoronaVac n, (%) | 25 (54.3%) | 5 (62.5%) |  |
| BBIBP-CorV + CoronaVac n, (%) | 3 (6.5%) | 0 (0.0%) |  |
| Days between the second inactivated SARS-CoV-2 vaccination and blood sampling | 30.0 (21-45) | 26.5 (21-40) | 0.055 |
| Days between the Influenza vaccination and second inactivated SARS-CoV-2 vaccination | **/** | 285.5 (191-314) | **/** |
| ^#^Presented as median (range). The Chi-Square statistic and Fisher’s exact test were used for categorical variables, and Mann-Whitney U test was used for continuous variables.  ALT, alanine aminotransferase; AST, aspartate aminotransferase; BMI, body mass index; HCs, healthy controls; PLT, platelet; RBC, red blood cell; TB, total bilirubin; WBC, white blood cell | | | |

| **Table S7. Correlation between clinical characteristics and anti-SARS-CoV-2 antibodies** | | | | | | | | |
| --- | --- | --- | --- | --- | --- | --- | --- | --- |
|  | Anti-RBD IgG | | | | Neutralizing antibody | | | |
|  | r | *P* | *β* | *P* | r | *P* | *β* | *P* |
| Age (years) | 0.172 | 0.150 |  |  | 0.084 | 0.482 |  |  |
| Gender (female) | -0.010 | 0.919 |  |  | 0.014 | 0.884 |  |  |
| BMI (kg/m^2) | -0.113 | 0.345 |  |  | -0.116 | 0.330 |  |  |
| RBC (10^9/L) | -0.113 | 0.345 |  |  | -0.134 | 0.262 |  |  |
| PLT (10^9/L) | 0.053 | 0.657 |  |  | 0.047 | 0.693 |  |  |
| WBC (10^9/L) | 0.012 | 0.921 |  |  | 0.020 | 0.867 |  |  |
| Lymphocyte (10^9/L) | 0.136 | 0.253 |  |  | 0.135 | 0.259 |  |  |
| ALT (U/L) | 0.175 | 0.142 |  |  | **0.212** | **0.073** | N/A |  |
| AST (U/L) | **0.258** | **0.028** | N/A |  | **0.321** | **0.006** | -0.036 | 0.774 |
| TB (μmol/L) | 0.149 | 0.212 |  |  | 0.174 | 0.144 |  |  |
| HBsAg (IU/mL) | -0.062 | 0.605 |  |  | 0.132 | 0.269 |  |  |
| HBeAg-positive | 0.063 | 0.515 |  |  | -0.029 | 0.766 |  |  |
| HBV DNA (IU/mL) | **-0.219** | **0.065** | -0.022 | 0.848 | **-0.202** | **0.089** | N/A |  |
| Antiviral treatment | **0.223** | **0.022** | 0.226 | 0.116 | **0.283** | **0.004** | 0.237 | 0.085 |
| Liver cirrhosis | **0.166** | **0.089** | -0.032 | 0.818 | **0.213** | **0.029** | 0.052 | 0.705 |
| SARS-CoV-2 vaccines (1=BBIBP-CorV, 2=CoronaVac, 3= BBIBP-CorV + CoronaVac) | **0.158** | **0.091** | 0.121 | 0.296 | 0.125 | 0.180 |  |  |
| Days between second dose of SARS-CoV-2 vaccine and blood collection | -0.035 | 0.769 |  |  | 0.052 | 0.666 |  |  |
| Influenza vaccination within 1 year before SARS-CoV-2 vaccination | **0.240** | **0.014** | **0.307** | **0.009** | **0.240** | **0.014** | **0.294** | **0.011** |
| Anti-H1N1 IgG Day 28 (μg/mL) | -0.214 | 0.610 |  |  | 0.048 | 0.911 |  |  |
| Anti-H3N2 IgG Day 28 (μg/mL) | -0.238 | 0.570 |  |  | -0.405 | 0.320 |  |  |
| Anti-B IgG Day 28 (μg/mL) | 0.405 | 0.320 |  |  | 0.571 | 0.139 |  |  |
| Spearman’s rank correlation (for two continuous variables) and Kendall’s rank correlation (when one of the variables was discontinuous) were used for two-variable correlation analysis. When the *P* value of a variable is lower than 0.1 (**bold**), this variable was included in multivariate linear regression analysis (enter method) for the further analysis. A two-sided *P* value lower than 0.05 was considered statistically significant (**bold**). r represents correlation coefficient, and *β* represents the standardized regression coefficient.  ALT, alanine aminotransferase; AST, aspartate aminotransferase; BMI, body mass index; HBeAg, hepatitis B e antigen; HBsAg, hepatitis B surface antigen; N/A, variables not included in multivariate linear regression analysis because of multicollinearity; PLT, platelet; RBC, red blood cell; RBD, receptor binding domain; TB, total bilirubin; WBC, white blood cell. | | | | | | | | |
